# Reminiscing and the Passage of Years: Investigating the Role of Affective Autobiographical Memories in Passage of Time Judgments

**DOI:** 10.3389/fpsyg.2021.713264

**Published:** 2021-09-27

**Authors:** Ferdinand Kosak, Christof Kuhbandner

**Affiliations:** Department of Psychology, University of Regensburg, Regensburg, Germany

**Keywords:** contextual change hypothesis, memory based approaches, passage of time judgments, subjective experience of time, storage size metaphor, autobiographical memory, fading affect bias

## Abstract

Previous research has shown that judgments of the experienced velocity of recent years passing by vary depending on the number of autobiographical memories being activated in the moment of judging. While a body of evidence shows affect to have an impact on both prospective and retrospective judgments on the experience of time for short periods, the effect of valence of memories on the experience of the passage of long intervals has not been examined yet. Thus, we asked 282 people to retrieve five either emotionally positive or negative memories from the last 5years before judging the subjectively experienced passage of time of these years. However, positive and negative events differ in some ways beyond valence, e.g., the ascribed impact on the participants’ subsequent lives as well as the stability of ascribed affective intensity: The latter decreased over time for negative but not for positive memories while ascribed impact was markedly higher for positive memories. Results indicate no significant differences between the two conditions, even after controlling for the aforementioned differences. However, exploratory analyses show that participants rate time to have passed faster, the longer the activated memories dated back on average, a result that seems in line with contextual-change hypothesis.

## Introduction

### Autobiographical Memory and the Experience of Time

A famous quote of Benjamin Franklin suggests that one should not squander time since it “is the stuff life is made of” (e.g., Leo-Lemay, 2006, p. 194). Although one could repulse the implicated utility-imperative, the simple truth of time being life’s basic commodity may explain why the perceived velocity of time is a phenomenon of high significance to humans. In fact, at least inhabitants of western countries seem to regularly claim that time flies by in everyday conversations.

This anecdotal observation gets evidential support by studies looking at passage of time judgments (POTJs) for longer intervals (ranging from days to 10years): when asked to judge the experienced velocity of these intervals, reported mean ratings consensually indicate a perception of time passing fast (e.g., Wittmann and Lehnhoff, 2005; Friedman and Janssen, 2010; Kosak et al., 2019).

Since the passage of years is obviously no sensorial experience, such judgments are necessarily based on derivations from other information. One possibility is that these reflect something like a cultural “meme” (see Lee and Janssen, 2019 for everyday theories concerning time experience) suggesting time to fly and the judgment itself being made without individuals actually examining their inner mental processes. If this was the only source, asking for POTJs could evoke nothing but the belief in this meme, and the complaint about time flying was just an empty phrase. However, ending the debate here would ignore the possibility that people might in fact be able to evaluate their experience of time passage: It has been argued that passage of time is actually the perception of succession, of one event happening after another (Deng, 2019). This can be read as a reflection of Franklin’s idea: in hindsight, life is the accumulation of time, which is mentally represented as succession of events. This view suggests that the information we use to both access and define time in the past are actually the memories of events from this exact past. Following these thoughts, trying to sense a subjective velocity of passed intervals means examining the succession and accumulation of accessible memories and – in order to be able to come to a judgment – compares the result with one’s individual conception of a prototypical interval.

**Figure 1:**
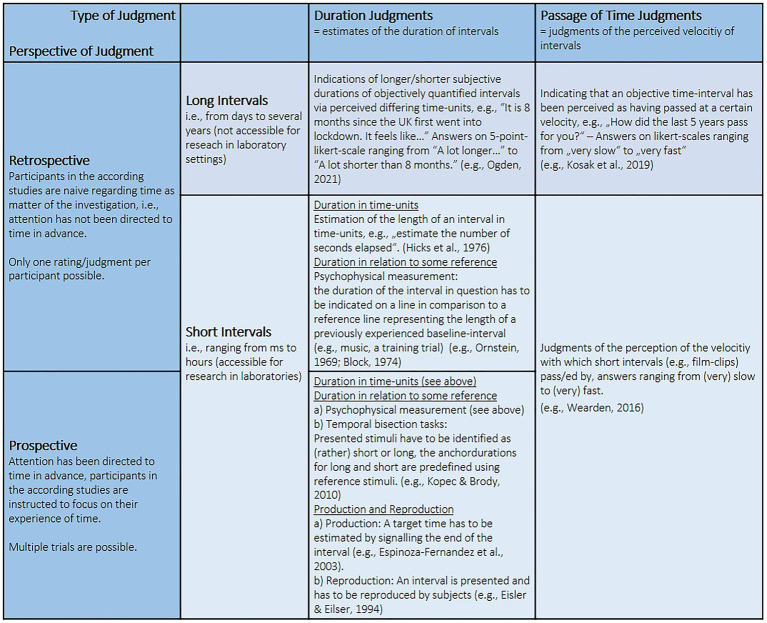
Typology of established paradigms regarding the subjective experience of time. Without claiming completeness, this comprehensive view captures and depicts some of the most common paradigms applied in psychological research investigating the experience of time. The differentiation between long and short intervals is a suggestion based on what length of intervals can or cannot be easily assessed in laboratories and therefore with the according paradigms.

These considerations are compatible with established theories explaining differences in retrospective timing (i.e., naïve reporting of time experience after an interval; see Figure 1 for a comprehensive view), namely, the storage size metaphor (Ornstein, 1969) and the contextual-change hypothesis (Block, 1985). The first claims that the higher the amount of information stored for the time interval in question, the longer it is subjective experience of time. This storage size, in turn, results from (1) the number of informational units encoded and (2) the complexity of the encoding process. The contextual-change hypothesis, by contrast, rejects the idea that the amount of stored information itself is decisive. Instead, it proposes that not all information is equally relevant and identifies the degree to which experienced information is perceived as indicating change as crucial (Block, 1985). This shifted the focus from the intervals content to the subjective interpretation of the information. Experimental studies investigating short intervals have confirmed this idea showing that (1) the level of processing (shallow vs. deep) leads to different memory content but not different estimates for duration and (2) given the same quantity of content, an experience of an irregular pattern indicates more contextual change and therefore an extended experience of time compared to a pattern with higher levels of routine (see, e.g., Block and Reed, 1978; Block, 1985).

Despite the differences regarding content itself or its perception being crucial, both approaches are based on the assumption that the information presented and stored from intervals are fundamental for the perception of time. Furthermore, this implies a “covert retrieval” (Block and Reed, 1978, p. 657), i.e., in the moment of judging time, people are not necessarily aware of this judgment being influenced by the information from the interval in question. This seems plausible for short intervals, typically investigated by using retrospective paradigms in laboratory settings, but assigning this hypothesis to longer intervals might be hasty. The retrieval of relevant memories – covert or not – to judge extensive parts of a life is obviously more complex than retrieving memories from a preceding interval ranging from seconds to minutes. In fact, due to the sheer amount of potential memories, a comprehensive evaluation of relevant information from long intervals is impossible. Thus, it seems reasonable to expect these judgments might depend on what is present in the moment of judging. This was confirmed in our previous study, where POTJs for the last 5years varied depending on the number of actively recalled memories from this interval: With a certain amount of memories being surpassed, POTJs were slower compared to a control condition with no explicit request to retrieve memories before providing POTJs. On the other hand, retrieving only few memories led to even faster POTJs compared to the control condition (Kosak et al., 2019).

### The Impact of Activated Information on Evaluations of Life

That salient information can affect supposedly extensive evaluations of life has been shown in another field of research, namely, wellbeing: A set of studies presented evidence showing that overall subjective wellbeing was rated higher after having recalled positive compared to negative autobiographical life events (Schwarz and Clore, 1983; Borg, 1987; Schwarz and Strack, 1999), although recent evidence suggests that effect sizes might have been overestimated in earlier studies (Yap et al., 2017). Similarly, having recalled one’s latest dating record (Strack et al., 1988) or verbally addressing the result of a preceding football game (Schwarz, 1987) had likewise consequences. In these studies, the direction of the shift in wellbeing depends on the affective valence of the salient information: overall life satisfaction was lower if negative information was activated, and *vice versa* regarding positive information. While this impact seems plausible given the coherence between the valence of memories and subjective wellbeing, a similar role of valence for the subjective experience of time seems not as obvious.

However, from an everyday perspective, common claims of time flying when one is having fun but dragging when experiencing boredom and/or discomfort suggest a possible importance of affect for the experience of time. The hereby-assumed mechanism seems plausible: Since situations perceived as negative are typically aversive, which implies a desire for the current situation to end or to improve, time has been suggested to be explicit in these situations. By contrast, in situations considered as positive, e.g., flow experiences, one’s awareness is usually led away from time so that time gets implicit (Fuchs, 2005). Thus, positive situations might often feel like having gone by (too) fast while experiencing something unpleasant or waiting in a boring situation is likely to feel dragging. This, however, applies primarily to prospective (i.e., participants being aware of time perception as matter of investigation; see Figure 1) judgments for a short-time perspective.

### The Role of Valence in Time Perception

In fact, a body of research covering prospective time perception for short intervals suggests that emotion has a significant impact on the experience of time (see Droit-Volet, 2019, for an overview) with some studies supporting the described association of valence and time perception. For instance, the presentation of positive stimuli was perceived as shorter than negative stimuli (e.g., Gable and Poole, 2012), and high levels of boredom lead to a relatively longer time perception (Danckert and Allman, 2005). Other studies show that stimuli of any valence can extend the subjective experience of time highlighting that the reason for differing results is a complex interplay of attention and arousal effects (e.g., Droit-Volet et al., 2004; Noulhiane et al., 2007; Lambrechts et al., 2011; Kliegl et al., 2015).

Little research covers the impact of emotional material and/or mood on retrospective judgments for longer intervals. One study reported no impact of film-induced affective states on the felt duration of a subsequent waiting-line situation (Chebat et al., 1995), while another reported that a wait with positive music was judged as longer than one with negative music, the duration of which was still overestimated compared to a no-music condition (Hui et al., 1997). Similarly, an entertaining situation (watching a movie) was judged as having lasted longer than a particularly boring task (waiting in an empty room, Wearden, 2005). Both experiments suggest not only that valence but also the content of the intervals in question matters, supporting the memory-based approaches’ main claim that the existence of more encoded and retrievable information leads to a lengthened experience of time in retrospect.

For the experienced passage of years, however, the role of affect has not been investigated yet. As mentioned before, a comprehensive retrieval of all potentially relevant information is obviously impossible, what explains the relevance of currently activated memories on POTJs (Kosak et al., 2019). However, whether the emotional valence of these memories is crucial remains an open question. From a theoretical perspective, the direction of a potential effect seems unclear: One plausible assumption suggests that the activation of negative events evokes and/or mirrors the experience of time dragging through the interval in question, potentially resulting in time being experienced as slow. At the same time, research has shown that negative memories are typically remembered in less detail and are less vivid compared to positive ones (D’Argembeau and Van der Linden, 2008; Sedikides and Skowronski, 2020). Thus, considering memory-based approaches, recalling positive memories could lead to more mental content compared to recalling the same number of negative events too. This would suggest that activating only negative memories should result in judging time as having passed faster compared to activating positive memories. To investigate whether POTJs differ depending on the valence of previously activated autobiographical memories, participants in the present study recalled either positive or negative personal memories from the last 5years before judging how fast these have passed for them.

## Materials and Methods

### Participants

Based on a power analysis (G^*^Power; Faul et al., 2007), aiming for 80% power for small to medium effects of *d*=0.35 (*p*<0.05), we targeted a minimum sample size of 260 participants. All data exclusions, manipulations, and measures used in the study are reported. Participants were recruited *via* Prolific,^1^ the Web site of the German edition of “Psychology Today,” and private sources. Participants on Prolific were compensated with 1,90 £, students of the Regensburg university received course credit, and all other participants received no compensation. 282 participants (149 *via* Prolific and 133 *via* other sources) recalled the required memories before providing POTJs and were included in the final sample. Mean age was 27.32years (*SD*=9.56), 33.3% were male, 62.8% female, and 3.9% did not disclose their gender. 52.4% of the participants were students, 37.3% employed, and 7.4% self-employed [the rest spread among other categories, e.g., household work, unemployed, or vocational training (multiple options could be selected)].

### Design and Procedure

The study was realized using the online-platform SoSci Survey (Leiner, 2019), participants spent *M*=15.96min (*SD*=5.56) to complete the questionnaire. When opening the survey, participants were introduced to the study without any information regarding time as a matter in its course. Instead, the study was presented as investigating the relation between subjective wellbeing and autobiographical memories. Additionally, participants were asked whether they had been diagnosed with post-traumatic stress disorder throughout the last 5years, suffer from flashbacks, intrusions, or dissociations, or have experienced things, of which the recall might cause strong and burdening emotional reactions. Answering yes to at least one of these questions ended the study, advising against taking part due to a potential confrontation with burdening memories. Otherwise, the next page provided detailed information about the survey and the use of data and finally, participants had to provide informed consent by ticking a checkbox.

The actual survey started with the satisfaction with life scale (Glaesmer et al., 2011) and the positive and negative affect scale (Krohne et al., 1996). Then, participants were randomly assigned to one of two experimental conditions and had to recall either five negative (*N*=137) or positive autobiographically relevant events (*N*=145) from the last 5years. The instruction was presented on a separate page, asking participants to look back on the past 5years and recall five positive/negative events from these years, which one has personally experienced as meaningful (German: “bedeutsam”) or defining (German: “prägend”). It was emphasized that what is meaningful or defining is a matter of each individual’s notion and that a keyword with a significant meaning only for the participant was sufficient. Each event was provided on a separate page with an input line. On the next two separate pages, subjects were asked to judge the POTJ for the last 5years and the last year (7-point Likert scales ranging from 1=very slow to 7=very fast). Subsequently, subjects were asked to briefly describe their thoughts when judging the passage of time before they had to recall another five memories of the opposite valence (this was done to avoid that participants of one group end the study with in-depth exposure to negative and burdening memories only). Then, all memories were presented to the subjects again asking them for some additional information, namely, the year of the event, emotional valence (1) at the time when the event took place (from here on referred to as “initial valence”) and (2) at the moment of retrieval (“current valence”), and impact on the subsequent life. Following these ratings, participants were asked to fill out a modified version of the Protestant work ethics scale (McHoskey, 1994) and a scale measuring time pressure (Friedman and Janssen, 2010). Finally, demographical information and a short evaluation of the questionnaire were requested.

### Qualitative Data

The qualitative data generated by asking participants about their thoughts when judging the passage of time was manually coded into the variable “thoughts while judging” using MAXQDA 2020 (VERBI Software, 2019). All participants referring explicitly to their past (e.g., “The events from the last years feel like being long ago but somehow still like yesterday,” Case 2,628) were coded with “memories” while participants whose statements did not touch on all memories (e.g., “Sometimes time goes by fast, sometimes slow, on average neither fast nor slow,” Case 2,472) were coded as “other.” Answers referring to high levels of routine and/or answers explicitly stating an absence of memories (e.g., “every day is just the same and eventless,” Case 2,727) were coded as “memories” as well, since these imply an attempt to recall memories.

### Ethics, Preregistration, and Repository

The study was conducted in accordance with the Declaration of Helsinki and the ethical guidelines of our university. In Germany, psychological studies of these type do not require ethical approval of an Ethics Committee.^2^ The study was preregistered using open science framework.^3^ All data and measurements including verbatim instructions regarding POTJs and the recall of autobiographical memories are accessible in a repository.^4^

## Results

A total of 79.9% of participants’ thoughts while judging the passage of time referred to memories (excluding participants that did not refer to memories did not lead to different results in the subsequently reported analyses). The two experimental conditions did not differ significantly regarding age, subjective wellbeing, time pressure, their values on the protestant work ethics scale (all *d*s<|0.13|, *p*s>0.31; see Table 1), or their thoughts while judging [*χ*^2^ (1, *N*=275)=1.31, *p*=0.25, *d*=0.14].

**Table 1:** Mean ratings on several variables reported by participants depending on experimental condition.

Variables	Positive before POTJ		Negative before POTJ	
	*M*	*SD*	*M*	*SD*
Age	27.63	9.79	27.01	9.33
SWLS	4.75	1.28	4.75	1.23
PANAS balance	1.06	1.18	1.05	1.14
POTJ5	5.41	1.27	5.25	1.08
POTJ1	5.74	1.39	5.82	1.27
Time pressure	4.95	1.33	4.79	1.30
PWE	2.74	0.51	2.75	0.52

SWLS refers to the satisfaction with life Scale (Glaesmer et al., 2011). PANAS balance reports the mean rating on negative items subtracted from the mean rating on positive items of the positive and negative affect scale (Krohne et al., 1996). POTJ5 and POTJ1 refer to the passage of time judgments for the last 5 years and the last year, respectively, measured with a 7-point Likert scale ranging from “very slow” to “very fast.” Time pressure is measured by a four item scale (7-point Likert) taken from Friedman and Janssen (2010). PWE = Protestant work ethic (19 items, 5-point Likert) reports the average on a slightly modified version of the according scale, taken from McHoskey (1994).

The average POTJs across experimental conditions were *M*=5.33 (*SD*=1.18) for the last 5years and *M=* 5.78 (*SD=* 1.33) for the last year.

Comparing the POTJs for the last 5years between the experimental conditions (positive vs. negative memories activated) revealed no significant overall differences [*t*(280)=1.17, *p*=0.241; *d*=0.136]. We further investigated the absence of a statistically significant mean difference in the frequentist framework by calculating a Bayes factor to relate evidence in favor of a null effect to evidence in favor of a non-null effect. The resulting Bayes factor of *BF*_10_=0.26 indicated moderate evidence in favor of the null (although relatively close to the range considered as weak, see, van Doorn et al., 2020). For the model, we used default Cauchy prior distributions with a scaling parameter of *r*=0.71. POTJs for the last year did not differ between conditions as well [*t*(280)=−0.50, *p*=0.616; *d*=−0.06].

With reference to potentially interacting variables, an interesting observation was that while the initially experienced affective intensity (i.e., valence ratings without direction, thus, ranging from 0=neutral to 3=very negative/positive) of the recalled events did not differ between conditions [*M*_positive_=1.97, *SD*=0.95, *M*_negative_=1.99, *SD=0*.69; *t*(253.06)=−0.17, *p*=0.87; *d*=0.02], the currently experienced affective intensity differed markedly [*M*_positive_=2.01, *SD*=0.72, *M*_negative_=0.74, *SD*=0.84; *t*(264.70)=13.37, *p*<0.001; *d*=1.62]. A mixed ANOVA with time (initial vs. current) and condition revealed both a significant effect of condition [*F*(1, 273)=53.65, *p*<0.001, *d*=0.91] as well as an interaction of condition and time [*F*(1, 273)=141.44, *p*<0.001, *d*=1.44], indicating that the affective intensity reported for the memories varied between “initial” and “current” depending on experimental condition. Thus, participants reported a strong decline of affective intensity for negative memories while intensity of positive memories did not change with time.

Furthermore, experimental conditions did not differ concerning the average time passed since the events [*M*_positive_=2.56, *SD*=0.70, *M*_negative_=2.64, *SD=0*.72; *t*(273)=0.26, *p*=0.35; *d*=0.11], while they varied markedly regarding the ascribed impact on the subsequent life [*M*_positiveMem_=5.92, *SD*=0.76, *M*_negativeMem_=3.78, *SD*=0.89; *t*(273)=21.30, *p*<0.001; *d*=2.57].

Regarding the specifications of the memories recalled (mean years since event, mean impact on subsequent life, and mean initial and current valence) before judging the passage of time, a linear regression reveals that two of these have a small but significant impact on POTJs for 5years: These were faster with a higher average of impact ascribed to the memories as well as with a longer average time passed since the events (see Table 2). Neither current nor initial affective intensity, however, was associated with POTJs.

**Table 2:** Linear model of memory specifications predicting passage of time for 5years.

Model	*b*	*SE B*	*β*	*p*
Constant	4.22	0.41		< 0.001
Avg. years since event	0.27	0.10	0.16	< 0.01
Impact on subsequent life	0.14	0.06	0.17	0.01
Current affective intensity	−0.03	0.08	−0.04	0.63
Initial affective intensity	−0.11	0.09	−0.08	0.21

*R*^2^=0.05, corrected *R*^2^=0.04, *p*=0.005. *Average. years since event*: for each event, the time passed since was selected from a dropdown menu, offering options from 1=less than 1year ago to 5=between 4 or 5years ago. All other items were rated on 7-point Likert scales: impact on subsequent life (1=very negative impact and 7=very positive impact) and initial affective intensity / current affective intensity: the emotional intensity at the time of the event taking place (initial, i.e., “How did you feel at the time the event had happened?”) and at the moment of filling out the questionnaire (current, i.e., “How do you feel now thinking back to that event?”). The original scales regarding valence were ranging from 1=very negative to 7=very positive. To compare the intensity, we transformed the scale into a unidirectional scale ranging from 0=not intensive at all to 3=very intense. Note that only specifications of memories activated before judging the passage of time had been included in the analysis.

Since impact on subsequent life does both differ between the experimental conditions and is associated with POTJs, we decided to control for potential interaction effects. Thus, we applied a linear mixed model including experimental condition, impact, and the interaction of condition and impact to predict POTJs for 5years (see, Hilbert et al., 2019). However, the overall model did not significantly explain variance of POTJs for 5years, *F*(271,3)=2.46, *p*=0.07, corrected *R*^2^=0.015 and neither condition nor impact nor the interaction of impact and condition did predict POTJs, all values of *p*>0.24. Additionally, we applied the same procedure to control for a potential impact of the fading of affective intensity on POTJs (e.g., the difference between the absolute values of initial and current intensity) but the model including condition, decline in affective intensity and the interaction of both did not explain any variance in POTJs; *F*(271,3)=0.56, *p*=0.64, corrected *R*^2^<0.01. These analyses show that none of the observed differences between positive and negative memories cover up a potential effect of valence on the experienced passage of time.

## Discussion

The main goal of this work was to investigate whether the emotional valence of currently activated memories has an impact on subsequent POTJs for intervals of several years. While the present study is the first investigating the impact of valence on retrospective POTJs for multiannual intervals, numerous studies have shown that emotional factors have a significant impact on prospective experience of time for short intervals (e.g., Droit-Volet et al., 2004; Noulhiane et al., 2007; Lambrechts et al., 2011; Kliegl et al., 2015), and some studies show an effect of emotional factors on the retrospective experience of time for periods up to some minutes (e.g., Hui et al., 1997; Wearden, 2005). However, our results show no differences in POTJs depending on the different emotional valence of currently activated memories, and this holds true after controlling for specifications that differ between positive and negative memories, such as currently experienced affective intensity, the fading of affective intensity, or the perceived impact of the memories on one’s subsequent life.

The last two aspects seem worth a closer look: Our results show that the relatively long intervals between the time the events took place and the recall lead to a lower affective intensity of retrieved negative compared to positive memories. This replicates the fading affect bias, i.e., the decline of affective intensity for negative but not positive memories (e.g., Skowronski et al., 2014). Consequently, negative memories are judged as less negative in the present than by the time the events took place. Other than, in studies investigating shorter intervals (i.e., the wait in Hui et al., 1997 or the movie excerpt in Wearden, 2005), by the time of judging the passage of time, the affective intensity of negative memories is systematically lower compared to positive ones. However, since the decline of intensity has no significant impact on POTJs and controlling for this decline does not uncover any differences between the experimental conditions regarding POTJs, it seems that valence of activated autobiographical memories simply does not affect POTJs for long intervals.

Another aspect that differs remarkably between positive and negative memories is the impact, people ascribed to the memories: Negative ones are reported to have had considerably less impact on the participants’ subsequent lives than positive memories. In the light of contextual-change hypothesis, which identified the perception of change as crucial for the subjective experience of time (Block and Reed, 1978), this would suggest that participants, which activated negative events before judging POTJs, should have rated time as having passed faster. However, across both conditions, a higher ascribed impact of the reported memories on subsequent life is associated with slightly faster POTJs. This seems surprising, but before voiding contextual-change hypothesis as an explanation for the perceived passage of years, one should note that participants had to recall specifically personally meaningful events. Presumably, this prompts the recall of memories that have had impact on participants’ lives so that the true variability of impact of past events might be considerably underestimated in our data. Additionally, the markedly lower impact ratings for negative compared to positive memories seem somewhat peculiar. In fact, it is difficult to come up with plausible explanations why negative life events, such as, for instance, severe illnesses or death of close persons, should have less impact on the subsequent life than positive events. One interpretation might come from the literature discussing the Pollyanna principle, describing a positivity bias in a range of cognitive phenomena including memory and judgment (Matlin, 2004). Drawing from these findings, a plausible mechanism seems that people tend to reduce the ascribed impact of negative events just in the way they leave behind negative affect while savoring positive affect (Walker et al., 2003). This cushioning of negative experiences may help staying mentally equipped for the challenges of life. However, confirmatory studies investigating both a lower ascribed impact of negative life events on the subsequent life itself or its interplay with the subjective experience of time would be necessary to support this interpretation.

In contrast, an exploratory finding in this study provides preliminary support for contextual change in experiences of long time intervals: The longer the reported memories dated back on average, the faster time was judged as having passed. In terms of contextual change, this might suggest that under the impression of little contextual changes in recent years, the time since is perceived as having passed comparatively fast. Given that the activation of memories has been shown to mitigate the experience of time passing fast (Kosak et al., 2019), prevention of time flying might be particularly effective when reminiscing in one’s recent past.

## Data Availability Statement

The raw data supporting the conclusions of this article is publicly available under https://osf.io/habrz/.

## Ethics Statement

Ethical review and approval was not required for the study on human participants in accordance with the local legislation and institutional requirements. The patients/participants provided their written informed consent to participate in this study.

## Author Contributions

FK and CK designed the study and analyzed the data. FK wrote the first draft of the manuscript. All the authors revised, read and approved the submitted manuscript.

## Conflict of Interest

The authors declare that the research was conducted in the absence of any commercial or financial relationships that could be construed as a potential conflict of interest.

## Publisher’s Note

All claims expressed in this article are solely those of the authors and do not necessarily represent those of their affiliated organizations, or those of the publisher, the editors and the reviewers. Any product that may be evaluated in this article, or claim that may be made by its manufacturer, is not guaranteed or endorsed by the publisher.

## Footnotes

^1^ www.prolific.co

^2^ https://www.dfg.de/foerderung/faq/geistes_sozialwissenschaften/

^3^ https://osf.io/v2ujc

^4^ https://osf.io/habrz/
